# Topical Astodrimer Sodium, a Non-Toxic Polyanionic Dendrimer, Demonstrates Antiviral Activity in an Experimental Ocular Adenovirus Infection Model

**DOI:** 10.3390/molecules26113419

**Published:** 2021-06-05

**Authors:** Eric G. Romanowski, Kathleen A. Yates, Jeremy R. A. Paull, Graham P. Heery, Robert M. Q. Shanks

**Affiliations:** 1 The Charles T. Campbell Ophthalmic Microbiology Laboratory, UPMC Eye Center, Department of Ophthalmology, University of Pittsburgh School of Medicine, Pittsburgh, PA 15213, USA; yateska@upmc.edu (K.A.Y.); shanksrm@upmc.edu (R.M.Q.S.); 2 Starpharma Pty Ltd., 4-6 Southampton Crescent, Abbotsford, VIC 3067, Australia; jeremy.paull@starpharma.com (J.R.A.P.); graham.heery@starpharma.com (G.P.H.)

**Keywords:** astodrimer sodium, SPL7013, dendrimer, antiviral, eye, adenovirus, EKC, animal model

## Abstract

There is no approved antiviral therapy for adenovirus (HAdV) ocular infections. Astodrimer sodium (SPL7013) is a polyanionic dendrimer with antiviral activity. The current study evaluated the ocular tolerability and anti-adenoviral efficacy of topical SPL7013 in rabbit ocular models. In a tolerability study, rabbits were treated with 3% SPL7013, vehicle, or 0.5% cidofovir. Their eyes were graded using the Draize scale. In antiviral efficacy studies, HAdV5 inoculated eyes were treated with 3% SPL7013, vehicle, or 0.5% cidofovir. Eyes were cultured for the virus on days 0, 1, 3, 4, 5, 7, 9, 11, and 14. Viral titers were determined. There were no differences in Draize scores between 3% SPL7013 and vehicle on any day. Cidofovir produced significantly higher Draize scores on day 12 than SPL7013 and vehicle. The 3% SPL7013 and 0.5% cidofovir significantly reduced daily viral titers and positive cultures per total compared with vehicle on several different days. The 3% SPL7013 and 0.5% cidofovir significantly reduced the duration of HAdV5 shedding compared to vehicle. The 3% SPL7013 demonstrated significantly more antiviral activity compared with vehicle in the Ad5/NZW rabbit ocular model. The 3% SPL7013 induced “minimal” to “practically non-irritating” Draize scores in the ocular tolerability study. Further development of astodrimer sodium as a topical antiviral therapy for adenoviral ocular infections is indicated.

## 1. Introduction

Ocular infections caused by human adenoviruses (HAdV) are among the most common ocular infections seen globally [1]. These infections manifest in three forms: epidemic keratoconjunctivitis (EKC), follicular conjunctivitis, and pharyngeal conjunctival fever. Current treatments are generally supportive as there is no approved or registered antiviral therapy for the treatment of adenoviral conjunctivitis.

Dendrimers are a novel class of nanoscale macromolecules that have been designed to interact polyvalently with a target and act as virucidal agents [2]. These precisely constructed macromolecules are characterized by multiple layers of branched subunits emanating from a central core and are constructed by the repeated stepwise addition of branched subunits to a reactive core [2]. An early generation dendrimer, SPL-2999, was shown to inhibit both HSV internalization into host cells and late-stage viral replication [3].

Subsequently, a newer dendrimer, astodrimer sodium (referred to herein as SPL7013), has been synthesized and has attracted increasing interest as an antiviral therapy [2,4,5,6,7,8]. SPL7013 is a dendrimer consisting of a divalent benzhydrylamine (BHA) core and four generations of lysine branches, with the outermost branches capped with naphthalene disulfonic acid groups that impart hydrophobicity and a high anionic charge to the dendrimer surface [4]. The highly charged surface of SPL7013 causes it to attach to targets on viruses, such as surface glycoproteins, thereby blocking viral attachment and/or adsorption to cells, which prevents infection [5]. SPL7013 has demonstrated in vitro virucidal activity against HSV, HIV, and SARS-CoV-2 [2,3,4,5,6,7].

SPL7013 was originally developed as a vaginal microbicide to prevent the transmission of HSV and HIV [8] and is now included as an antiviral agent in the lubricant of registered condom products in Europe, Japan, and Australia. SPL7013 produces low cytotoxicity in vitro and minimal vaginal irritation in animal models [5] and has proven effective in preventing the vaginal transmission of SHIV in pigtailed macaques [9]. Phase 1, 2, and 3 clinical trials of VivaGel^®^ (1% and 3% SPL7013, Starpharma Pty Ltd.) demonstrated that the agent is safe for vaginal use in women [10,11,12,13,14,15]. These trials, and the demonstration of the antibacterial properties of SPL7013, led to the registration of VivaGel^®^ with indications for the treatment and prevention of bacterial vaginosis in Europe, Australia, New Zealand, and multiple countries in Southeast Asia, the Middle East, and Africa [14,15].

Preclinical in vitro data with SPL7013 demonstrated antiviral activity against adenoviruses (unpublished data, Starpharma personal communication). This finding led to the current study in which topically applied SPL7013 was evaluated for ocular tolerability in naïve rabbit eyes and antiviral activity against adenoviruses in an Ad5/NZW rabbit ocular model. The NZW rabbit antiviral efficacy model has been used to evaluate several potential anti-adenoviral agents including topical cidofovir, which has demonstrated anti-adenoviral activity in several preclinical studies [16,17,18,19,20,21,22,23]. Cidofovir was used as a positive control for in vivo antiviral activity in this study. The results of this study demonstrate that topical 3% SLP7013 is non-toxic to rabbit eyes and possesses antiviral activity against HAdV5 in the Ad5/NZW rabbit ocular model.

## 2. Results

### 2.1. Ocular Tolerability Study

**Figure 1:**
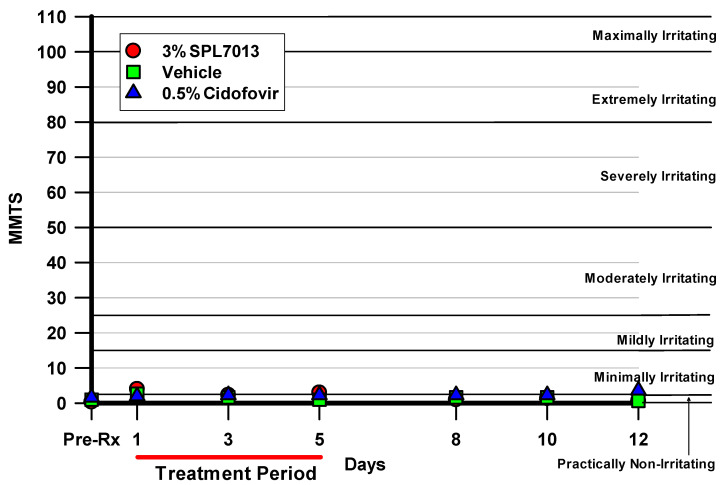
The MMTS values from each treatment group and observation day. 3% SPL7013 demonstrated scores in the minimally irritating to practically non-irritating categories. There were no significant differences among the treatment groups for any day other than day 12 where 3% SPL7013 (median score 0.0) and vehicle (median score 0.0) had significantly lower scores than 0.5% cidofovir (median score 4.0) (*p* ≤ 0.0278, K-W). The red line indicates the treatment period (Days 1–5).

Naïve NZW rabbits were topically treated in both eyes with 3% SPL7013, vehicle, or 0.5% cidofovir 4 times daily for 5 consecutive days (3% SPL7013 and vehicle) or twice daily for 5 consecutive days (0.5% cidofovir). The eyes were graded using the Draize scale scoring system [24] on days 1, 3, 5, 8, 10, and 12 after treatment on those days on which treatment was performed. Figure 1 demonstrates the maximum mean total scores (MMTS values) [25] from each treatment group and observation day. Treatment with 3% SPL7013 produced scores in the minimally irritating to practically non-irritating categories for all days. There were no significant differences in the Draize scores among the treatment groups for any day other than day 12 where 3% SPL7013 (median score 0.0) and vehicle (median score 0.0) had significantly lower scores than 0.5% cidofovir (median score 4.0) (*p* ≤ 0.0278, K-W).

### 2.2. Antiviral Efficacy Study

This antiviral efficacy study was performed using the Ad5/NZW rabbit ocular replication model which only evaluates viral replication. Ocular clinical signs of infection are not demonstrated in this model and were not evaluated. NZW rabbits were topically inoculated in both eyes with HAdV5 following corneal scarification. The following day, the rabbits were divided into three topical treatment groups: (1) 3% SPL7013 (8X daily for 4 days then 4X daily for 6 days); (2) vehicle (8X daily for 4 days then 4X daily for 6 days); and (3) 0.5% cidofovir (2X daily for 7 days). The treatments were applied to both eyes. Ocular swabbing to recover adenovirus from the tear film and corneal and conjunctival surfaces was performed on day 0, 3 h following inoculation, and on days 1, 3, 4, 5, 7, 9, 11, and 14 after inoculation.

**Figure 2:**
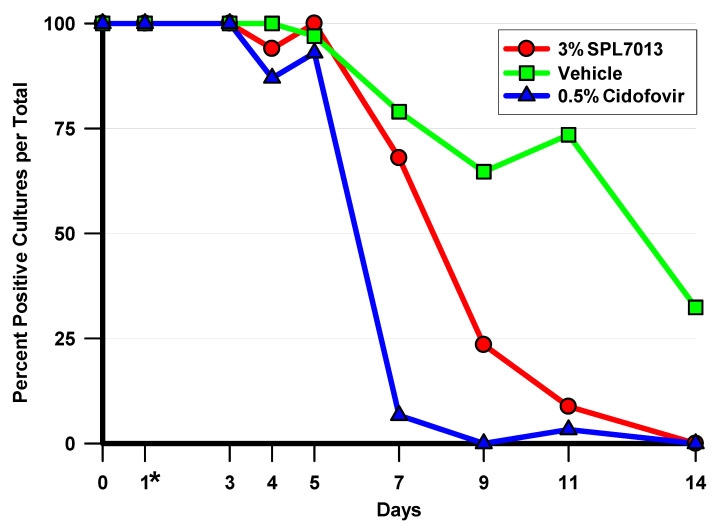
The percentage of HAdV5 positive cultures per total cultures over the course of the antiviral efficacy study. Significant differences were demonstrated on day 7 (0.5% Cidofovir < 3% SPL7013 = Vehicle), day 9 (0.5% Cidofovir < 3% SPL7013 < Vehicle), and days 11 and 14 (0.5% Cidofovir = 3% SPL7013 < Vehicle) (*p* ≤ 0.005, chi-square). <, significantly less than; =, statistically indistinguishable; *, indicates that treatment was initiated on day 1.

The percentage of HAdV5-positive cultures per total for each culture day is presented in Figure 2. Any ocular culture with measurable HAdV5 PFU was considered a positive culture. This was the most stringent of the viral outcomes measured in this study as one virus plaque was considered a positive culture. Treatment with 3% SPL7013 significantly decreased the number of HAdV5-positive cultures per total cultures on days 9, 11, and 14 compared to vehicle (*p* ≤ 0.001, chi-square (C-S)), while 0.5% cidofovir significantly decreased the number of HAdV5-positive cultures per total cultures on days 7, 9, 11, and 14 compared to vehicle (*p* < 0.001, C-S. In addition to this, the treatment with 0.5% cidofovir significantly decreased the number of HAdV5-positive cultures per total cultures on days 7 and 9 compared to the treatment with 3% SPL7013 (*p* ≤ 0.005, C-S).

**Figure 3:**
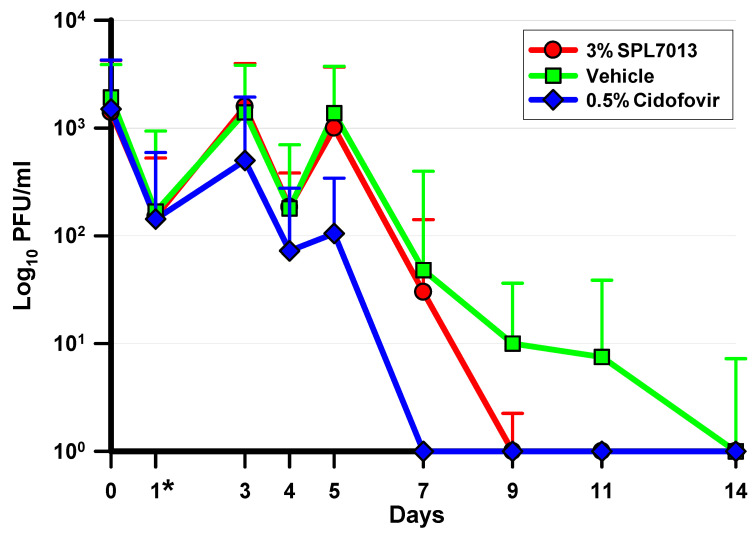
The median and interquartile ranges of ocular HAdV5 titers over the course of the antiviral efficacy study. Significant differences were demonstrated on days 5 and 7 (0.5% Cidofovir < 3% SPL7013 = vehicle) and on days 9, 11, and 14 (0.5% Cidofovir = 3% SPL7013 < Vehicle) (*p* ≤ 0.0001, Kruskal–Wallis ANOVA). <, significantly less than; =, statistically indistinguishable; *, indicates that treatment was initiated on day 1.

The median daily ocular HAdV5 titers are presented in Figure 3. These data were analyzed using the Kruskal–Wallis ANOVA with Dunn’s multiple comparisons test. Treatment with 3% SPL7013 produced significantly lower HAdV5 titers on days 9, 11, and 14 compared with vehicle (*p* ≤ 0.0001, K-W). Treatment with 0.5% cidofovir produced significantly lower viral titers on days 5, 7, 9, 11, and 14 compared with vehicle (*p* ≤ 0.0001, K-W) and on days 5 and 7 compared with 3% SPL7013 (*p* < 0.0001, K-W).

The duration of HAdV5 shedding in each eye was determined by the final day on which the eye had a positive HAdV5 culture. This outcome measure represents the length of the infection in the eye. Treatment with 3% SPL7013 (median 7 days) and 0.5% cidofovir (median 5 days) significantly shortened the duration of HAdV5 shedding compared with vehicle (median 11 days) (*p* ≤ 0.005, K-W). The 0.5% cidofovir treatment significantly shortened the duration of HAdV5 shedding compared with 3% SPL7013 (*p* = 0.017, K-W).

## 3. Discussion

The search for antiviral therapies for adenoviral ocular infections is ongoing. The search started with traditional nucleoside analog antiviral agents [26]. Nucleoside analogs are antiviral agents that inhibit a critical component of the viral replication process within a host cell. Several of these nucleoside analog antiviral agents, such as cidofovir, ddC, and filociclovir, have shown antiviral activity against HAdV5 when administered topically to the eye in the Ad5/NZW rabbit ocular model [16,17,18,19,20,21,22,23]. To date, none of these antiviral therapies have gained FDA or EMA approval to treat adenoviral ocular infections.

Another antiviral strategy for the treatment of adenoviral ocular infections has emerged using antiseptic-type antiviral agents. Several antiviral agents have recently been tested in clinical trials for these infections. NVC-422 (AL-46383A, auriclosene), povidone–iodine, and a combination of povidone–iodine, and dexamethasone have recently been evaluated in clinical trials for viral conjunctivitis [27]. These agents inactivate viruses on the ocular surface but have no effect on intracellular viruses. The limitation of these agents is the short residence time on the ocular surface, which in turn requires frequent topical doses to produce antiviral efficacy [27]. Currently, none of these antiseptic-type antiviral agents have gained US FDA or EMA approval for use.

Dual intracellular and extracellular mechanisms of action would be advantageous for an antiviral agent. The dual interaction could limit intracellular viral replication and also inactivate the virus on contact once it is released from the host cell, as long as it is present on the ocular surface. This inactivation would prevent additional cells from becoming infected, thus limiting the infection. Dendrimers have been shown to have a predominantly extracellular mechanism of action [5] but may also have intracellular antiviral activity [3]. It is unclear if the intracellular antiviral effect of dendrimers is the result of a direct or indirect mechanism of action. Therefore, the exact mechanism is unknown [3].

The current study evaluated the topical ocular tolerability and in vivo antiviral efficacy of the dendrimer astodrimer sodium (SPL7013) against an experimental ocular adenoviral infection. Topical 3% SPL7013 instilled 4 times daily for 5 days was well tolerated in the eyes in the ocular tolerability study, producing practically non-irritating to minimally irritating Draize scores during the treatment period, and practically non-irritating Draize scores during the week following treatment.

The antiviral efficacy study used a treatment regimen for the 3% SPL7013 of 8 times per day for the first 4 days, and then 4 times daily for 6 days. This treatment regimen produced a loading dose of the SPL7013 early in the infection to maximize its antiviral efficacy during the time of peak adenoviral replication in the eyes. This 3% SPL7013 treatment regimen significantly shortened the length of the infection by 4 days compared to vehicle-treated eyes. A decrease in the length of the infection along with the corresponding decreases in positive cultures and viral titers can potentially decrease patient signs of infection and possibly decrease the number of vision-altering subepithelial corneal infiltrates that may form in patients.

SPL7013 did not produce significant decreases in viral replication during the early part of the infection, as was seen with the 0.5% cidofovir treatment (days 5 and 7), but it did produce significant reductions in the HAdV5 titers and positive cultures per total cultures during the latter part of the infection (days 9, 11, and 14) compared with the vehicle control. It has been previously suggested that extracellular, surface acting, antiseptic type antiviral agents may not reduce viral titers during the peak of viral replication in the rabbit models, but they may shorten the length of the HAdV5 infection [27]. This effect is accomplished by inactivating free viruses on the ocular surface, thereby eliminating the potential for those virus particles to infect naïve cells, prolonging the viral replication, and limiting the infection [27]. It appears that the antiviral efficacy produced by 3% SPL7013 is consistent with this profile, suggesting that the primary antiviral mechanism of action of topical SPL7013 in the Ad5/NZW rabbit ocular model is extracellular.

In this study, we evaluated 3% SPL7013 formulated in a proprietary aqueous vehicle that was optimized for SPL7013. We chose not to formulate the 0.5% cidofovir in this vehicle as its antiviral activity had not been tested in this vehicle. Furthermore, we wanted to compare the antiviral activity of 3% SPL7013 in its optimized vehicle compared to 0.5% cidofovir formulated in IV saline, which has demonstrated reproducible antiviral activity in our previous studies [16,17,18,19,20,21,22,23].

Based on the data presented from this study, we conclude that 3% SPL7013 is non-toxic to rabbit eyes and has anti-adenoviral activity. Further refinement and optimization of the dosing regimen may be required before progressing to human clinical trials for viral conjunctivitis. Continued development of SPL7013 (astodrimer sodium) for the treatment of adenoviral ocular infections is indicated.

## 4. Materials and Methods

### 4.1. Experimental Compounds

The formulated 3% SPL7013 and its vehicle (formulation without SPL7013) were provided by Starpharma Pty Ltd., 4-6 Southampton Crescent, Abbotsford, Victoria 3067, Australia. The vehicle is a proprietary aqueous formulation that contains standard ophthalmic excipients including a viscosity modifier, preservative, humectant, antioxidant, and pH modifiers. The 0.5% cidofovir was prepared in IV saline from the 7.5% injectable form of cidofovir (Vistide, Gilead Sciences, Inc., Foster City, CA, USA or Cidofovir Injection, Mylan Institutional LLC, Rockford, IL, USA).

### 4.2. Adenovirus and Cells

A clinical adenovirus isolate of type 5 (HAdV5) was collected anonymously from a patient presenting with adenoviral conjunctivitis at the Charles T. Campbell Ophthalmic Microbiology Laboratory at the UPMC Eye Center, University of Pittsburgh, Pittsburgh, PA, USA. This isolate was retrieved from a frozen retrospective clinical collection of de-identified adenovirus isolates stored for diagnostic test validations. The HAdV type of the isolate was determined using serum neutralization.

A549 human lung carcinoma cells were grown and maintained in Eagle’s MEM supplemented with 10% fetal bovine serum (Sigma Cell Culture Reagents, St. Louis, MO, USA). This cell line was used to prepare the adenovirus stock and for the determination of the viral titers in the Ad5/NZW rabbit ocular replication model.

### 4.3. Animals

Two- to three-pound female NZW rabbits were obtained from either Robinson Services, Inc. Mocksville, NC, USA or Harlan, Indianapolis, IN, USA. All the animal studies conformed to the ARVO Statement on the Use of Animals in Ophthalmic and Vision Research. Approval was obtained from the University of Pittsburgh IACUC (Protocols #13082349 and #14063973) prior to the initiation of the study. All the institutional and USDA guidelines regarding animal experimentation were followed.

### 4.4. Ocular Tolerability Study Experimental Design

A total of 9 NZW rabbits were divided into 3 topical treatment groups (*n* = 3): (1) 3% SPL7013, (2) vehicle and (3) 0.5% cidofovir. Rabbits were treated topically with 37 µL drops in both eyes 4 times daily for 5 consecutive days (days 1–5) for 3% SPL7013 and vehicle, and twice daily for 5 consecutive days (days 1–5) for the 0.5% cidofovir group. The regimen chosen for the SPL7013 and vehicle groups matches a commonly used ophthalmic standard regimen, whereas the cidofovir regimen of 0.5% twice daily is the regimen used in our many experimental studies [17,18,19,20,21,22,23]. The rabbits’ eyes were graded in an unmasked fashion using the Draize scale [24] on day 1 before treatment was initiated and on days 1, 3, 5, 8, 10, and 12 during and after treatment. The maximum mean total score (MMTS) was calculated and categorized as previously described [25].

### 4.5. Antiviral Efficacy Study Experimental Design

This study was performed using a total of 49 rabbits. The rabbits were anesthetized systemically with intramuscular injections of 40 mg/kg ketamine (Ketathesia™, Henry Schein^®^ Animal Health, Dublin, OH, USA) and 4 mg/kg xylazine (X-Ject E, Henry Schein^®^ Animal Health, Dublin, OH, USA), and treated topically on the cornea with 0.5% proparacaine (Proparacaine Hydrochloride Ophthalmic Solution, USP 0.5%, Akorn, Inc., Lake Forest, IL, USA). Once fully anesthetized, both corneas of the rabbits were topically inoculated with 50 µL (1.5 × 10^6^ PFU/eye) of HAdV5 in both eyes after corneal scarification with 12 cross-hatched strokes of a #25 sterile needle. The rabbits’ eyelids were closed, and their eyes were rubbed for 5 s to ensure the contact of the viral inoculum to the entire ocular surface. Twenty-four hours later, the rabbits were arbitrarily assigned to one of three topical treatment groups: (1) 3% SPL7013 (*n* = 17); (2) vehicle (*n* = 17); or (3) 0.5% cidofovir (*n* = 15). Rabbits were treated topically in both eyes with 37 µL drops, 8 times daily for 4 days (days 1–4), then 4 times daily for 6 days (days 5–10) for groups 1 and 2. The 0.5% cidofovir rabbits were treated twice daily for 7 days (days 1–7). Ocular swabbing to recover adenovirus from the tear film and corneal and conjunctival surfaces was performed on day 0, 3 h following inoculation, and on days 1, 3, 4, 5, 7, 9, 11, and 14 after inoculation, at least 1h after the final antiviral dose. The swabs were placed into 1 mL of tissue culture medium and frozen at −80 °C pending plaque assay.

### 4.6. Determination of Ocular Viral Titers (Plaque Assay)

The ocular culture samples to be enumerated for viral counts were thawed, diluted, and inoculated onto A549 cell monolayers. The samples were adsorbed onto the monolayers for 3 h with intermittent shaking, after which the cells were overlaid with tissue culture media containing 0.5% methylcellulose. After 7 days of incubation at 37 °C and 5% CO_2_, the cells were fixed and stained with a 0.5% gentian violet-containing formalin. The number of viral plaques was counted under a dissecting microscope. The HAdV5 titers were then calculated and expressed as plaque-forming units per milliliter (PFU/mL).

### 4.7. Statistical Analysis

The graded tolerability data were analyzed using Kruskal–Wallis (K–W) ANOVA with Dunn’s multiple comparisons (GraphPad Prism, San Diego, CA, USA). Ocular titer data were analyzed using Kruskal–Wallis (K–W) ANOVA with Dunn’s multiple comparisons (GraphPad Prism) and chi-square (C-S) (Minitab, State College, PA, USA). Significance was established at the *p* ≤ 0.05 confidence level.

## Data Availability

The data reported in this study are available in this manuscript.
